# Effect of Growth Mindset on School Engagement and Psychological Well-Being of Chinese Primary and Middle School Students: The Mediating Role of Resilience

**DOI:** 10.3389/fpsyg.2016.01873

**Published:** 2016-11-29

**Authors:** Guang Zeng, Hanchao Hou, Kaiping Peng

**Affiliations:** ^1^Department of Psychology, Tsinghua UniversityBeijing, China; ^2^Research Center of Positive Psychology, Tsinghua UniversityBeijing, China

**Keywords:** positive education, growth mindset, resilience, school engagement, psychological well-being, Chinese primary school students, Chinese middle school students

## Abstract

The objective of positive education is not only to improve students’ well-being but also their academic performance. As an important concept in positive education, growth mindset refers to core assumptions about the malleability of a person’s intellectual abilities. The present study investigates the relation of growth mindsets to psychological well-being and school engagement. The study also explores the mediating function of resilience in this relation. We recruited a total of 1260 (658 males and 602 females) Chinese students from five diversified primary and middle schools. Results from the structural equation model show that the development of high levels of growth mindsets in students predicts higher psychological well-being and school engagement through the enhancement of resilience. The current study contributes to our understanding of the potential mechanisms by which positive education (e.g., altering the mindset of students) can impact psychological well-being and school engagement.

## Introduction

Positive education is “education for both traditional skills and for happiness” (Seligman et al., 2009, p. 293). Peterson (2006) put forward the idea that schools could become positive institutions, and placed great emphasis not only on the academic performance of students but also on their character and well-being. Positive education teaches the skills of well-being through direct practice and the curriculum, aiming to equip students with skills to build their resilience, optimism, character strengths, formation of positive relationships, and other significant factors that contribute to a flourishing life. In the literature, there are several benefits of positive education, including providing an antidote to the depression of young teenagers, serving as a pathway which increases life satisfaction, nurtures creativity, and fosters class cohesion and civic citizenship (Seligman et al., 2009; Kern et al., 2015). Notably, research shows that positive education promotes the psychological characteristics and character strengths which are associated with students’ higher academic performance, lower risk behaviors, and long-term benefits for their physical health (Caprara et al., 2000; Durlak et al., 2011). However, to the best of our knowledge, little research has directly investigated the “why” – underlying mechanisms involved in cultivating the psychological characteristics and character of students that can increase both psychological well-being and school achievement. To date, there is no study that demonstrates the causal relationship between psychological well-being and school performance. In particular, we do not know whether positive education enhances school performance by increasing the sense of psychological well-being, or whether psychological well-being results from improved school performance. Or alternatively, is there an additional, underlying mechanism that improves both? The present study addresses these questions.

### Growth Mindset, Psychological Well-Being, School Engagement

#### Growth Mindset

Growth mindsets, also known as implicit theories, are defined as core assumptions about the malleability of personal qualities (Dweck and Leggett, 1988; Dweck et al., 1995; Molden and Dweck, 2006; Yeager and Dweck, 2012). Students hold different implicit theories, from a more fixed mindset or entity theory of intelligence to of a more growth mindset or incremental theory. Fixed mindset students “see intellectual ability as something of which people have a fixed, unchangeable amount,” while growth mindset students “see intellectual ability as something that can be grown or developed over time” (Yeager and Dweck, 2012, p. 303). The mindsets of students make them perceive their academic world differently. The growth mindset promotes resilience while the fixed one does not (Dweck et al., 1995; Dweck, 2006). Students with a fixed mindset tend to conceive everything as a measurement of their ability and intellect, such as academic performance, challenges, troubles, etc. However, students with a growth mindset tend to think of their academic lives in terms of learning, growing, and developing. Growth mindset students interpret setbacks, challenges, and effort as effective approaches to improving their ability, intelligence, and experience.

#### Psychological Well-Being

Diener et al. (2009) has advanced a new form of well-being, named psychological well-being, representing optimal human positive functioning. Psychological well-being can be assessed by the Psychological Well-Being Scale (PWB), which includes all important components of well-being such as meaning and purpose, engagement and interest; supportive and rewarding relationships; contributing to the well-being of others, competency, self-acceptance, optimism, and being respected (Diener et al., 2009).

#### School Engagement

School engagement is a positive, fulfilling and study-related state of mind characterized by vigor, dedication, and absorption (Schaufeli et al., 2002a). Specifically, vigor is described as a high level of energy and mental resilience when studying; dedication refers to a sense of significance, enthusiasm, inspiration, pride and challenge; absorption means concentration and happiness when performing one’s studying tasks (Schaufeli et al., 2002b). In the literature, research shows the relationships between school engagement and adolescent students’ characteristics, such as school drop-out (Finn and Rock, 1997), substance use (Bond et al., 2007), mental health (Bond et al., 2007), and academic achievement (Marks, 2000; Wang and Holcombe, 2010). Research also has found that a high level of school engagement during upper secondary school predicts success in students’ educational transition after upper secondary school (Vasalampi et al., 2009). In addition, Nurmi and Salmela-Aro (2002) found that school engagement and absence of burnout lay the foundation for successful educational decisions and trajectories. School engagement is found to be associated with academic outcomes such as achievement and high school completion (Dotterer and Lowe, 2011).

### The Present Study

In the literature, research shows that growth mindset can lead to school achievement. There are many intervention experiments that demonstrate that changing students’ theories of intelligence from a fixed mindset to a growth mindset exerts impact on their academic behaviors in the long run (Aronson et al., 2002; Good et al., 2003; Blackwell et al., 2007). Aronson et al. (2002) found that African American students who were encouraged to view intelligence as malleable reported greater enjoyment of the academic process, greater academic engagement, and obtained higher grade point averages (GPA) than their counterparts in two control groups. Research also found that increasing the growth mindset of students helps to raise their math grades more in low-achieving seventh-grade students than high-achieving students (Good et al., 2003; Blackwell et al., 2007).

Brooks and Goldstein (2001) defined resilience as the capacity to cope effectively with past and present adversity. Notably, Ryff et al. (1998) also proposed that resilience is the capacity to maintain and recover their high well-being in the face of life adversity. Connor and Davidson (2003) show that resilience acts as a protective factor in facing negative consequences and therefore aids individuals in maintaining their physical and psychological well-being. Previous studies demonstrate that resilience contributes to the well-being of students (Ryff and Singer, 1996, 2000; Ryff et al., 1998). Yeager and Dweck (2012) contend that the underlying mechanism of growth mindset that leads to academic achievement seems to rely on the goals of students, their beliefs about effort and their attributions about their setbacks, and learning strategies in the face of academic difficulties, which are effective ways to promote resilience.

Burnette et al. (2013) suggest that growth mindset can increase the resilience level of students in such a way that growth mindset interventions help students understand academic challenges in a way that promotes learning and resilience. Fixed mindset students perceive academic challenges as signs of lack of intelligence, which diminishes the resilience of students in academic areas, even for high-achieving students (Dweck et al., 1995; Dweck, 2006). Notably, even when students were taught skills to be resilient in school, they may not apply these skills adequately because of their fixed mindset (Blackwell et al., 2007). The growth mindset students, meanwhile, interpret the academic challenges as a chance to improve their ability and sharpen their learning skill, which contributes to their resilience in academic areas, no matter for high or low achieving students (Hong et al., 1999; Blackwell et al., 2007; Nussbaum and Dweck, 2008). Additionally, growth mindset students were more resilient and earned higher grades when they confronted challenging school transitions (Blackwell et al., 2007). Therefore, it seems that resilience is a potential factor that plays an important role in the psychological mechanisms relating growth mindset to academic achievement.

Taken together, based on the cited research, growth mindset can be seen as a precursor of resilience, psychological well-being and school engagement. This seems to suggest that the growth mindset, resilience, all have independent impacts on psychological well-being and school engagement. However, none of the previous studies actually demonstrated empirically that resilience has a mediating effect.

Accordingly, the current study tests the relationships between growth mindset, school engagement, and psychological well-being. Importantly, the present study also investigates the underlying mechanism by which the growth mindset leads to school engagement as well as psychological well-being. It is our hypothesis that (a) the growth mindset will be positively related to the resilience in primary and middle school students; (b) growth mindset will predict students’ school engagement and psychological well-being; and (c) resilience plays a mediating role between growth mindset and psychological well-being and school engagement.

## Materials and Methods

### Participants

This study gathered convenience samples from five schools located in Guangdong province, China. The present study employed a diversified sample in order to increase the generalizability of the study. The five schools represent a wide variety of school types in the province, including two primary schools (one public and one private school), two middle schools (one top public and one averaged public school) and one vocational middle school. The school administrations of each participated school chose 5–6 classes (around 45 students in each class) from their school to join this research project. There are 26 classes in total in the current research.

A total of 1279 students participated in the survey. Nineteen students were excluded from the analysis because of missing data in their surveys, so the quantity of valid respondents was 1260 (658 males and 602 females). The average age of these respondents was 13.49 years, ranging from 7 to 20 years (*SD* = 3.20). The demography of each school was presented in **Table 1**.

**Table 1:** Demography of the respondents in the five schools.

School	Male	Female	Age (Mean)	Age (*SD*)
(1) Primary school A	140 (54.1%)	119 (45.9%)	10.13	1.44
(2) Primary school B	146 (54.1%)	124 (45.9%)	10.90	1.99
(3) Middle school A	138 (48.6%)	146 (51.4%)	17.12	1.03
(4) Middle school B	169 (64.3%)	94 (35.7%)	13.21	0.99
(5) Vocational middle school	65 (35.3%)	119 (64.7%)	16.82	1.08
Total	658 (52.2%)	602 (47.8%)	13.49	3.20

### Measures

The measures of growth mindset, resilience and psychological well-being were translated into Chinese by two translators, and were then back-translated by two other translators, which ensured that the meanings of the Chinese version are in line with the English version. The measure of school engagement is the Chinese version of Utrecht Work Engagement Scale-student (UWES-S) which has been revised and published in China (Fang et al., 2008). All scales were scored so that higher scores represented higher levels of the variable.

#### Growth Mindset

The Growth Mindset Inventory (Dweck, 2006) was used to measure the degree of the growth mindset of responders. The Chinese version of the scale consists of four items (e.g., No matter who you are, you always can change your intelligence a lot). Responses were made on a 6-point Likert-type scale. The reliability for this measure was α = 0.74.

#### Resilience

The Brief Resilience Scale (BRS), which was developed by Smith et al. (2008), was used to measure participants’ resilience. The Chinese version of the scale includes three items (e.g., I tend to bounce back quickly after hard times). Responses were made on a 5-point Likert-type scale. The reliability for this measure was α = 0.70.

#### Psychological Well-Being

The eight-item Flourishing Scale, which was called PWB (Diener et al., 2010) was employed to measure participants’ PWB. One Chinese version of this scale has been reported to have high reliability (α = 0.91), and high validity in a sample of undergraduate and postgraduate students (Lin, 2015). Researchers translated it again for primary and middle school students. The scale includes eight items. For example: I lead a purposeful and meaningful life. Responses were made on a 7-point Likert-type scale. The reliability for this measure in this study was α = 0.92.

#### School Engagement

Utrecht Work Engagement Scale-student which was originally developed by Schaufeli et al. (2002a,b) and revised into Chinese by Fang et al. (2008), was used to assess participants’ school engagement. UWES-S includes three subscales: vigor (six items; e.g., At my class, I feel that I am bursting with energy), dedication (five items; e.g., I find the studies that I do full of meaning and purpose) and absorption (six items; e.g., Time flies when I’m studying). Responses were made on a 7-point Likert-type scale. The reliability for each subscale was as follows: vigor (α = 0.87), dedication (α = 0.85) and absorption (α = 0.92).

### Procedure

This research received the approval from the Human Research Ethics Committee of Tsinghua University. Our research group also obtained the consent of the school administration, teachers and students. Before the application of the questionnaires, the participants were informed about the objectives of this research project, and confirmed that all data would be kept confidential, only accessible to the research group and be only used for research purposes. The data of the study variables were collected in the course of regular school hours. The online survey link was sent to the school administrations in the five participating schools. The teachers of the participating classes organized their students, and brought them to their school computer rooms to complete the survey.

### Data Analysis

Data analysis subsequently included two procedures. First, statistical descriptions and Pearson correlations were calculated through the SPSS 19.0. Second, structural equation modeling with latent variables (SEM) was adopted to analyze mediation effects using the Mplus 7 (Byrne, 2013). The item responses have been treated as continuous variables, and ML (maximum likelihood) was used as the estimator. This decision is based on the article (Rhemtulla et al., 2012) suggesting that ML is recommended when there are five or more points in Likert-scales. Also, ML is fairly robust and, therefore, recommended for slightly non-normal data (skewness < 2 and kurtosis < 7) (Finney and DiStefano, 2006). In this study, skews ranged from -1.13 to 0.26, and kurtoses ranged from -1.38 to 0.25, satisfying the condition.

A series of indicators were used to evaluate model fit, including chi-square (χ^2^), the comparative fit index (CFI), the Tucker Lewis index (TLI), the standard root mean square residual (SRMR) and the root mean square error of approximation (RMSEA) (Schreiber et al., 2006). Although the χ^2^ statistic is often reported, other indicators are commonly used to determine how well the model fits (Byrne, 2013, p. 69). According to Hu and Bentler (1999), the model fits well when CFI > 0.95, TLI > 0.95, SRMR < 0.08, RMSEA < 0.06.

## Results

The relations between the variables included in the model, as well as the descriptive statistics, are shown in **Table 2**, which shows that all the variables, including students’ growth mindset, resilience, psychological well-being and school engagement, were significantly and positively related.

**Table 2:** Association among study measures.

	1	2	3	4	5	6	7
(1) Growth Mindset	—						
(2) Resilience	0.22^∗∗^	—					
(3) Psychological Well-Being	0.22^∗∗^	0.36^∗∗^	—				
(4) School Engagement	0.24^∗∗^	0.29^∗∗^	0.56^∗∗^	—			
(5) Engagement – Vigor	0.22^∗∗^	0.23^∗∗^	0.51^∗∗^	0.97^∗∗^	—		
(6) Engagement – Dedication	0.25^∗∗^	0.31^∗∗^	0.57^∗∗^	0.96^∗∗^	0.89^∗∗^	—	
(7) Engagement – Absorption	0.24^∗∗^	0.30^∗∗^	0.57^∗∗^	0.98^∗∗^	0.93^∗∗^	0.91^∗∗^	—
*M*	12.59	19.83	42.69	78.03	26.74	23.47	27.83
SD	3.10	4.59	11.13	25.27	9.12	7.68	9.23

*^∗∗^*p* < 0.01.*

### Measurement Model

**FIGURE 1:**
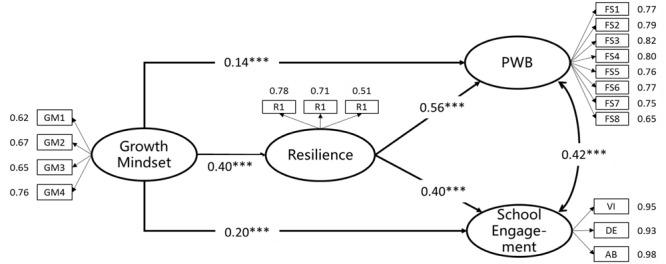
**Results of SEM (standardized estimates for statistically significant paths) for growth mindset, resilience, PWB and school engagement.** VI, vigor; DE, dedication; AB, absorption. ^∗∗∗^*p* < 0.001.

Structural equation modeling with latent variables (SEM) was used to test the hypothesis model that growth mindset predicts PWB and school engagement directly, and via resilience indirectly. Growth mindset was represented by four items, resilience was represented by three items and PWB was represented by eight items. School engagement was assessed by three subscales: vigor, dedication and absorption. The model for these analyses is depicted in **Figure 1**.

Fit indices were satisfactory, χ^2^(129) = 703.97, *p* < 0.01, CFI = 0.96, TLI = 0.95, SRMR = 0.04, RMSEA = 0.06. Loadings of the 18 observed indicators on the relevant latent construct were all in the predicted directions and all loadings were significant. As expected, growth mindset positively predicted PWB (β = 0.14, *p* < 0.001) and school engagement (β = 0.20, *p* < 0.001).

### Mediation by Resilience

Bootstrapping procedure (Preacher and Hayes, 2008) was conducted to examine the hypothesis that resilience mediates the effects of growth mindset on PWB and school engagement. Results show that the indirect effects from growth mindset to PWB and school engagement through resilience were significant (0.22, *p* < 0.001 for PWB and 0.16, *p* < 0.001 for school engagement). The 95% confidence intervals of the indirect effect estimate for PWB was 0.17 -0.27, and for school engagement was 0.12 -0.20.

### Analysis of Different Age Groups

To explore whether the model fits across ages, researchers tested the hypothetical model on different age groups. All students were divided into three age groups by their age, including those aged under 12, aged between 13 and 15, and aged 16 and over. For each age group, SEM was conducted to test the model fit of different age groups.

Result shows that the mediation model fits well in different age groups (see **Table 3**). The effects of different pathways, including direct, indirect and total effects of growth mindset on psychological well-being and school engagement, were included in **Table 3** as well. All pathways are significant, except the direct effects of growth mindset on psychological well-being and school engagement in the 13-to-15 age group. The direct effects of growth mindset on psychological well-being and school engagement in this group are marginally significant (*p* = 0.060 and *p* = 0.054, respectively).

**Table 3:** Analysis of different age groups.

	Age group			
	All	≤12	13–15	≥16
**Demography**				
Male	658 (52.2%)	296 (54.2%)	163 (63.7%)	199 (43.4%)
Female	602 (47.8%)	250 (45.8%)	93 (36.3%)	259 (56.6%)
Total	1260	546	256	458
Age(Mean)	13.49	10.45	13.50	17.10
Age(*SD*)	3.20	1.67	0.65	0.81
**Model statistics**				
χ*2*	703.968	378.847	327.437	410.97
*Df*	129	129	129	129
RMSEA	0.059	0.060	0.078	0.069
CFI	0.959	0.958	0.934	0.947
TLI	0.952	0.951	0.921	0.937
SRMR	0.037	0.038	0.056	0.049
**Model pathways**				
GM→BRS	0.396^∗∗∗^	0.323^∗∗∗^	0.464^∗∗∗^	0.486^∗∗∗^
BRS→PWB	0.556^∗∗∗^	0.622^∗∗∗^	0.472^∗∗∗^	0.509^∗∗∗^
BRS→SE	0.403^∗∗∗^	0.434^∗∗∗^	0.333^∗∗∗^	0.388^∗∗∗^
PWB→SE	0.421^∗∗∗^	0.381^∗∗∗^	0.466^∗∗∗^	0.449^∗∗∗^
GM→PWB (direct)	0.142^∗∗∗^	0.119^∗∗∗^	0.150^∗^	0.171^∗∗^
GM→SE (direct)	0.200^∗∗∗^	0.251^∗∗^	0.156^∗^	0.166^∗∗^
GM→BRS→PWB (indirect)	0.220^∗∗∗^	0.201^∗∗∗^	0.219^∗∗∗^	0.248^∗∗∗^
GM→BRS→SE (indirect)	0.160^∗∗∗^	0.140^∗∗∗^	0.155^∗∗∗^	0.189^∗∗∗^
GM→PWB (total)	0.362^∗∗∗^	0.320^∗∗∗^	0.370^∗∗∗^	0.419^∗∗∗^
GM→SE (total)	0.359^∗∗∗^	0.391^∗∗∗^	0.310^∗∗∗^	0.355^∗∗∗^

*^∗^*p* ≤ 0.10, ^∗∗^*p* ≤ 0.05, ^∗∗∗^*p* ≤ 0.001. GM, growth mindset; BRS, resilience; PWB, psychological well-being and SE, school engagement.*

## Discussion

Positive education aims to both improve well-being and academic achievement. However, little is known about the psychological mechanism that underpins these relationships. The current study was designed to obtain better insight into the possible associations between growth mindset, psychological well-being, and school engagement, with special attention to the possible role of resilience as mediator.

The result of the current study supports our previous hypothesis that the growth mindset is positively associated with resilience, school engagement and psychological well-being. Results from the structural equation model (SEM) show that developing high levels of growth mindsets in students predicts higher psychological well-being and school engagement through the enhancement of resilience. Additionally, given that the mental landscape of students has a tendency to change as they grow older, we examined whether this SEM fit for different age cohorts. In particular, the current study divided the participating students into three age groups according to their school levels, including those aged under 12 (primary school), aged between 13 and 15 (junior high school), and aged 16 and over (high school or vocational school). For each age group, SEM was conducted to test the model fit of different age groups. Result shows that all pathways of different age cohorts in the structural equation model are statistically significant, implying that the mediation model fits well in different age groups. Importantly, we must point out that the direct effects of growth mindset on psychological well-being and school engagement in the 13-to-15 age group are marginally significant (*p* = 0.060 and *p* = 0.054, respectively). One possible explanation might be related to the relatively smaller sample size of the 13-to-15 age group (*N* = 256), compared to other two groups – aged under 12 (*N* = 546) and aged 16-or-over age group (*N* = 458).

To the best of our knowledge, this full model of the relations between growth mindset, psychological well-being, school engagement and resilience has never been tested. However, several of the paths have been examined separately and the causal relationships have been established by longitudinal studies and experimental studies in the laboratory. In the literature, research has found that (a) higher resilience leads to higher overall well-being (Karreman and Vingerhoets, 2012; Lü et al., 2014); (b) greater resilience contributes to school engagement and academic performance (Aronson et al., 2002); (c) growth mindset contributes to self-regulation and goal achievement (Burnette et al., 2013). (d) growth mindset is moderately correlated with overall well-being, using the PERMA measurement for adults’ well-being (Kern et al., 2015) and EPOCH measurement for adolescents’ well-being (Kern et al., 2016). In addition, the current finding supports the previous studies that resilience contributes to the well-being of students (Ryff and Singer, 1996, 2000; Ryff et al., 1998). Moreover, the growth mindsets can predict the school engagement and academic performance. Our finding supports previous studies showing that students who hold a growth mindset and endorse a strong incremental theory of intelligence, will outperform those who hold a fixed mindset and adopt a more entity theory of intelligence (Blackwell et al., 2007).

As hypothesized, SEM analysis demonstrated that resilience functioned as a partial mediator between growth mindset and psychological well-being and school engagement. In particular, resilience serves as a mechanism to explain why growth mindset students are both high in school achievement and psychological well-being. The current research shows that students with growth mindset are more likely to bounce back from setbacks in academic and learning tasks, and in turn be more engaged in their schoolwork. When primary and middle school students hold the belief that their intelligence and ability is changeable are more resilient, this serves as a protective factor that enables students to adaptively cope with their highly competitive and stressful learning environment and effectively go through the hardships and obstacles of academic and daily life. Therefore, students with a growth mindset have better psychological well-being and are more likely to engage in schoolwork, than students who think their intelligence is fixed and unchangeable.

Because resilience plays a partial instead of a full mediator role between growth mindset, psychological well-being and school engagement, it implies that there may be other possible intervening variables. For instance, Blackwell et al. (2007) proposed an intervening pathway that an incremental theory of intelligence leads to positive efforts, beliefs, and learning goals, which in turn lead to fewer ability-based, helpless attributions and more positive strategies, which in turn lead to enhancement of test scores. Further research is needed to investigate the possible intervening variables.

### Limitations

There are several limitations of the current study that should be considered. First, the mediation model was examined by a cross-sectional designed study, which prevents us from drawing causal conclusions. Future study should utilize prospective and longitudinal approaches to demonstrate the causal relationships between the study variables. Second, since the current study employs the questionnaires as its primary measurement method, the common method variance could be a concern. Although instructions prior to the questionnaires in the current study that made it clear that there were no right or wrong answers helped to reduce the bias of common method variance, future studies could apply other procedural efforts to diminish this risk, such as varying scale types, positive and negative item wordings, and simple and concrete questions (Podsakoff et al., 2012).

Third, school engagement has been shown to moderately correlate with academic achievement (Wang and Holcombe, 2010; Dotterer and Lowe, 2011). It is ideal to measure actual academic achievement, such as grades and test scores of entrance examinations, as outcome variables. However, such information is not available for the current study. Future studies may benefit from getting such information as measurements for students’ academic achievement. In addition, future studies can combine the self-report of psychological well-being with objective, physical measurements such as health medical records and stress hormones to get a more comprehensive perspective on students’ overall well-being.

Fourth, the current study investigates how growth mindsets contribute to well-being. However, as well-being is not the opposite of negative outcomes, such as depressive symptoms, dysfunctional behaviors, and other psychological problems (Bech et al., 2003), future studies should also investigate the possibility that fixed mindsets predict the negative outcome variables. It is also reasonable to infer that the growth mindset can serve as a protective factor against psychological problems, such as depression, behaviors problems, school disengagement, burnout, and other negative outcome variables.

## Conclusion

In conclusion, the current study aims to investigate the possible mediating roles of resilience in the associations between growth mindset, psychological well-being, and school engagement. Resilience acts as a partial mediator between growth mindset, psychological well-being and school engagement, implying that resilience might be the key factor in reaching the objective of positive education, not only to enhance the well-being of students but also their academic achievement.

## Ethics Statement

Research Ethics Committee in psychology Department, Tsinghua university. We used online questionnaires to invest student’s psychological constructs, which has no harmful impacts on students. We also follow the informed consent discipline, which means that we told every student’s the information about this research, and invite them to participate in. If they do not want to participate, they can refuse to answer the questionnaires. Most of them agreed. No vulnerable populations were involved.

## Author Contributions

GZ and HH designed experiments; GZ and HH carried out experiments; HH analyzed experimental results. GZ and HH wrote the manuscript. KP supervised the experimenting, analyzing and writing process.

## Conflict of Interest Statement

The authors declare that the research was conducted in the absence of any commercial or financial relationships that could be construed as a potential conflict of interest.
